# Acute Gastroenteritis on Cruise Ships — Maritime Illness Database and Reporting System, United States, 2006–2019

**DOI:** 10.15585/mmwr.ss7006a1

**Published:** 2021-09-24

**Authors:** Keisha A. Jenkins, George H. Vaughan, Luis O. Rodriguez, Amy Freeland

**Affiliations:** ^1^Vessel Sanitation Program, Water, Food Environmental Health Services Branch, National Center for Environmental Health, CDC; ^2^Commissioned Corps, U.S. Public Health Service

## Abstract

**Problem/Condition:**

Gastrointestinal illness is common worldwide and can be transmitted by an infected person or contaminated food, water, or environmental surfaces. Outbreaks of gastrointestinal illness commonly occur in crowded living accommodations or communities where persons are physically close. Pathogens that cause gastrointestinal illness outbreaks can spread quickly in closed and semienclosed environments, such as cruise ships. CDC’s Vessel Sanitation Program (VSP) is responsible for conducting public health inspections and monitoring acute gastroenteritis (AGE) illness on cruise ships entering the United States after visiting a foreign port.

**Period Covered:**

2006–2019.

**Description of System:**

VSP maintains the Maritime Illness Database and Reporting System (MIDRS) for monitoring cases of AGE illness among passengers and crew sailing on cruise ships carrying ≥13 passengers and within 15 days of arrival at U.S. ports from foreign ports of call. Cruise ships under VSP jurisdiction are required to submit a standardized report (24-hour report) of AGE case counts for passengers and crew 24–36 hours before arrival at the first U.S. port after traveling internationally. If the cumulative number of AGE cases increases after submission of the 24-hour report, an updated report must be submitted no less than 4 hours before the ship arrives at the U.S. port. A special report is submitted to MIDRS when vessels are within 15 days of arrival at a U.S. port and cumulative case counts reach 2% of the passenger or crew population during a voyage. VSP declares an outbreak when 3% or more of the passengers or crew on a voyage report AGE symptom to the ship’s medical staff.

**Results:**

During 2006–2019, a total of 37,276 voyage reports from 252 cruise ships were submitted to MIDRS. Of the 252 cruise ships, 80.6% were extra large in size (60,001–120,000 gross registered tons [GRT]), 37.0% and 32.9% had voyages lasting 3–5 days and 8–10 days, respectively, and 53.2% were traveling to a port in the Southeast region of the United States at the time the final MIDRS report was submitted. During 2006–2019, VSP received 18,040 (48.4%) 24-hour routine reports, 18,606 (49.9%) 4-hour update reports, and 612 (1.6%) special reports (2% and 3% AGE reports). Incidence rates decreased from 32.5 cases per 100,000 travel days to 16.9 for passengers and from 13.5 to 5.2 for crew. Among passengers, AGE incidence rates increased with increasing ship size and voyage length. For crew members, rates were significantly higher on extra-large ships (19.8 per 100,000 travel-days) compared with small and large ships and on voyages lasting 6–7 days. Geographically, passenger incidence rates were highest among ships underway to ports in California, Alaska, Texas, New York, Florida, and Louisiana. Among passengers, AGE incidence rates were significantly higher on ships anchoring in California (32.1 per 100,000 travel-days [95% confidence interval (CI) = 31.7–32.4]); among crew, they were significantly higher in the South region of the United States (25.9 [CI = 25.1–26.7]).

**Interpretation:**

This report is the first detailed summary of surveillance data from MIDRS during 2006–2019. AGE incidence rates decreased during this time. Incidence rates among passengers were higher on mega and super-mega ships and voyages lasting >7 days. AGE incidence among crew was higher on extra-large ships and voyages lasting 6–7 days. Ship size and voyage length are associated with AGE incidence rates, and more targeted effort is needed to prevent disproportionate AGE incidence rates among passengers and crew sailing in high-risk situations.

**Public Health Actions:**

Maritime AGE surveillance provides important information about the epidemiology of gastrointestinal illness among cruise ship populations traveling in U.S. jurisdictions. AGE illness is highly contagious and can be transmitted quickly within vessels. State and local public health departments in the United States can use data in this report to better inform the traveling public about the risk for AGE and the importance of their role in minimizing the risk for illness while traveling onboard cruise ships. Key elements for reducing exposure to AGE illness, limiting the spread of illness, and preventing AGE outbreaks are proper hand hygiene practices and prompt isolation of symptomatic persons. Passengers can work in collaboration with cruise lines to promote onboard public health by frequently washing their hands, promptly reporting AGE illness symptoms, and isolating themselves from other persons immediately after illness onset. Access to and proper use of handwashing stations can reduce the risk for illness transmission aboard cruise ships.

## Introduction

Acute gastroenteritis (AGE) illnesses affect millions of persons worldwide (*1*,*2*) (https://www.cdc.gov/norovirus/trends-outbreaks/worldwide.html). The most common modes of transmission are through interaction with an infected person, consumption of contaminated food and water, and contact with contaminated environmental surfaces (*1*,*2*). Outbreaks of AGE illness commonly occur in crowded living accommodations or communities where persons are crowded together and can spread quickly in semienclosed environments, such as cruise ships. The Vessel Sanitation Program (VSP) is responsible for conducting environmental health inspections and monitoring the occurrence of AGE illness on cruise ships entering the United States from a foreign port.

Since 1975, VSP has worked collaboratively with the cruise ship industry to monitor AGE illness and minimize the risk for communicable diseases onboard cruise ships (*3*). In the early 1970s, cruise ships experienced an excessive number of gastrointestinal disease outbreaks, which led CDC to establish VSP in 1975 (*4*). The mission of VSP is to prevent and control the introduction, transmission, and spread of AGE illnesses on cruise ships to U.S. ports from cruise ships sailing from foreign ports. However, VSP does not monitor AGE illness on cargo ships and private yachts. VSP is authorized under the Public Health Service Act (42 U.S.C. Section 264 *Quarantine and Inspection Regulations to Control Communicable Diseases*) to take necessary measures to prevent the introduction and transmission of communicable disease into the United States from a foreign country (*5*).

The two main operational components of VSP are unannounced environmental health ship inspections and surveillance and outbreak investigations of AGE. Federal regulation 42 (CFR Section 71.41 *General Provisions, Foreign Quarantine Requirements Upon Arrival at U.S. Ports: Sanitary Inspections*) allows VSP to conduct unannounced ship inspections to identify the existence of pests, contaminated food or water, and other unsanitary conditions that could lead to the spread of communicable diseases (*6*).

VSP obtains its operating funds through user fees paid by the cruise lines after ships undergo unannounced environmental health inspections. Every ship under VSP jurisdiction is subject to twice-yearly inspections to ensure the ship maintains an appropriate level of sanitation (*7*). Cruise ship companies pay an inspection fee based on each ship’s gross registered tonnage (GRT) (https://www.cdc.gov/nceh/vsp/desc/about_inspections.htm). Inspection scores range from 0–100. An inspection score lower than 86 is considered unsatisfactory and subjects the ship to an unannounced reinspection.

VSP actively monitors the occurrence of AGE illness as directed by regulation 42 CFR Section 71.41(c), which specifies that the master of a ship carrying ≥13 passengers must report the number of AGE cases (including zero) among passengers and crew and record it in the ship’s medical log during the current cruise (*7*). Since April 1, 1975, VSP has maintained records of diarrheal illness for passenger ships visiting U.S. ports (*4*). Until 2001, ship masters reported the number of diarrheal illnesses by radio or telephone to CDC’s VSP 24 hours before arrival at a U.S. port. Ship masters no longer submit gastrointestinal illness (which includes diarrhea and other symptoms) reports by radio. Currently, illness counts are submitted by telephone, fax, or email or directly to the Maritime Illness Database and Reporting System (MIDRS).

This report uses MIDRS data to describe the incidence rates of AGE illness onboard passenger cruise ships that travel from foreign countries by ship size, voyage length, and regional U.S. ports of call for arrival. This is the first comprehensive summary of surveillance data to describe AGE incidence trends for cruise ships sailing in U.S. jurisdiction. State and local public health departments in the United States can use data in this report to better inform the traveling public about the risk for AGE and the importance of their role in minimizing the risk for illness while traveling onboard cruise ships.

## Methods

 VSP monitors cases of AGE illness among passengers and crew sailing on cruise ships carrying ≥13 passengers and within 15 days of arrival at a U.S. port from a foreign port of call. MIDRS was created in 2000 to allow submission of AGE reports to VSP through a web-based system. MIDRS is a syndromic surveillance system that collects cumulative case counts of passengers and crew reporting gastrointestinal symptoms to the ship medical staff during a single voyage. Each report submitted to MIDRS includes cruise ship name, voyage number, embarkation and disembarkation dates and locations, total number of passengers and crew, and number of passenger and crew that reported to the medical clinic with specific AGE symptoms (i.e., vomiting, diarrhea, abdominal cramps, headache, myalgia, or fever defined as ≥100.4°F [≥38°C]). AGE reports in MIDRS are submitted through the web-based portal system, email, fax, or phone call. Ships might submit multiple MIDRS reports per voyage, and each report is a cumulative count of ill passengers and crew that report to the medical center with AGE symptoms. Because final voyage case counts are not collected in MIDRS, data in this report were based on the last cumulative voyage report submitted to MIDRS. Although AGE outbreaks are stratified by passenger and crew, the occurrence of outbreaks is not mutually exclusive (i.e., not limited to a single population) for passengers and crew. The total number of AGE outbreaks will include overlap among these populations; however, for this report, outbreaks were categorized as passenger- or crew-associated outbreaks if >3% of the crew or passenger population reported AGE illness (VSP’s outbreak threshold).

### MIDRS Reporting

Cruise ships under VSP jurisdiction are required to submit a standardized AGE report (24-hour report) of case counts for passengers and crew 24–36 hours before arrival at a U.S. port after traveling internationally. If the cumulative number of AGE cases changes after submission of the 24-hour report, a 4-hour update report must be submitted no less than 4 hours before the ship arrives at the U.S. port. For voyages lasting >15 days, reporting of cumulative AGE case counts is required for cases that occur 15 days before the expected arrival at a U.S. port. When vessels are within 15 days of arrival at a U.S. port and cumulative case counts reach 2% of the passenger or crew population, a special report is submitted to MIDRS, which initiates active monitoring of illness patterns onboard the ship. MIDRS data do not reflect final AGE case counts at the time of disembarkation because ships are not required to report final voyage case counts for passengers and crew. VSP defines an outbreak as a cumulative case count that meets or exceeds 3% of the passenger or crew population and, in response, VSP conducts a remote environmental and epidemiologic consultation or an onboard investigation when logistically feasible (https://www.cdc.gov/nceh/vsp/desc/about_investigations.htm).

### Reportable Case Definition

In 1975, a reportable AGE case was limited to diarrheal illness defined as a person experiencing three or more watery stools in a 24-hour period. In 2000, the definition of a reportable AGE case was expanded to include a person who experienced three or more loose stools within a 24-hour period or vomiting plus one other symptom (i.e., diarrhea, abdominal cramps, headache, myalgia, or fever defined as ≥100.4°F [≥38°C]) in a 24-hour period. The revised case definition was first published in the 2000 VSP Operations Manual and became fully operational in 2001 (*8*). In 2011, VSP modified the definition of a reportable AGE case to include the frequency of diarrheal episodes “above normal for the individual” to be consistent with the World Health Organization’s definition of diarrhea (*9*) (https://www.who.int/news-room/fact-sheets/detail/diarrhoeal-disease).

### Data Analysis

The period covered in this surveillance analysis began in 2006 because it was the first year cruise ships were required to provide a unique voyage number, which provided the ability to differentiate multiple voyage reports submitted by the same ship each year. This analysis includes all AGE case reports submitted to VSP during 2006–2019 from cruise ships carrying ≥13 passengers and entering the United States from foreign ports of call. AGE incidence rates per 100,000 travel days and trends were examined for various cruise ship and voyage characteristics, including ship size (GRT), voyage length (days), and regional port it was traveling to at the time the MIDRS report was submitted (grouped into seven regions defined by VSP). Categorization of ship size was based on VSP’s categories used for the program’s construction and inspection activities: extra small, small, and medium (≤30,000 GRT), large (30,001–60,000 GRT), extra large (60,001–120,000 GRT), mega (120,001–140,000 GRT), and super mega (>140,001 GRT). Voyage length was categorized to match cruise line marketing travel packages: 3–5 days, 6–7 days, 8–10 days, and 11–14 days. This report excludes voyages lasting <3 days and >21 days, and vessels that carried <100 passengers. Regional ports and regions were categorized as follows: Northwest (Washington, Oregon, and Alaska), Hawaiian Islands and Guam, California, South (all ports on the Gulf of Mexico excluding Florida), Northeast (all states north of and including North Carolina), Southeast (all Atlantic ports in Florida, Georgia, and South Carolina), and Caribbean Islands. Poisson regression was used to examine rates, with travel days as an offset variable, and rate trends, with calendar year as the predictor variable. Wald chi-square was used to assess the association between variables, with the significance threshold set at 0.05. Rates and incidence trends were examined by traveler type (passenger and crew) and cruise ship characteristics (ship size based on GRT and voyage length in days). Reference groups for comparing incidence rates were based on cruise ship characteristic categories with the largest total population. Regional AGE incidence rates were mapped using natural breaks as cut points, and port-specific AGE incidence rates (total population, passengers, and crew) were mapped using quartile breaks as cut points to examine the variability around the median rate. Ports with <20 reports were considered unstable, and no data are presented. All statistical analyses were conducted using SAS software (version 9.4; SAS Institute).

## Results

During 2006–2019, a total of 37,258 AGE surveillance reports were obtained from 252 cruise ships arriving at U.S. ports (Table 1). Among the reports received, 48.4% (n = 18,040) were 24-hour reports, 49.9% (n = 18,606) were 4-hour reports, and 1.6% (n = 612) were special reports. Of the 252 cruise ships, 80.6% were extra large. Approximately 37.0% had voyages lasting 3–5 days and 32.9% had voyages lasting 8–10 days. The majority of the cruise ships (53.2%) were traveling to a port in the Southeast region at the time the report was submitted.

**TABLE 1:** Number and percentage of passenger cruise ships* sailing from foreign to U.S. ports, by selected characteristics — Maritime Illness Database and Reporting System, United States, 2006–2019

Characteristic	No. (%)^†^
No. voyage reports (unduplicated)	37,258 (100)
No. ships (unduplicated)	252 (100)
**Report type^§^**	
24-hour	18,040 (48)
4-hour	18,606 (50)
Special	612 (2)
**Ship size (gross registered tons)^¶^**	
Extra small, small, medium (≤30,000)	1,500 (4)
Large (30,001–60,000)	4,510 (12)
Extra large (60,001–120,000)	30,039 (81)
Mega (120,001–140,000)	917 (3)
Super mega (≥140,001)	292 (1)
**Voyage length (days)^¶^**	
3–5	13,772 (37)
6–7	6,031 (16)
8–10	12,239 (33)
11–14	3,111 (8)
15–21	2,105 (6)
Regional port of arrival**	
California	5,021 (14)
Caribbean Islands	2,267 (6)
Hawaiian Islands	250 (1)
Northeast	3,756 (10)
Northwest	3,384 (9)
South	2,767 (7)
Southeast	19,813 (53)

* Cruise ships carrying 13 passengers and within 15 days of arrival at a U.S. port from a foreign port of call.

† Sum might not total 100% because of rounding.

^§^ 24-hour report = case counts for passengers and crew 24–36 hours before arrival at a U.S. port; 4-hour report = changes in cumulative case counts since submission of the 24-hour report must be submitted no less than 4 hours before the ship arrives at U.S. port; Special report = cumulative AGE case counts ≥2% of passengers or crew.

^¶^ Categorization of ship size is based on Vessel Sanitation Program (VSP) categories used by VSP for the program’s construction and inspection activities, and voyage length is categorized to match cruise line marketing travel packages.

** **Northwest** (WA, OR, AK): ADK, AKU, ANC, AOR, ATT, BAK, BWA, COR, DHA, EFC, FHW, GLB, HAK, HNS, HOM, IAK, JNU, KIS, KOD, KTN, MET, NOM, PAN, POR, PTB, SEA, SGY, SIT, SWD, VDZ, WAI, WRG, WTR, YAK; **Hawaiian Islands, Guam, American Samoa, Saipan**: GUA, HIL, HNL, KAH, KAU, KON, LAH, MAU, PAS, SAI, SAM; **California**: ACA, CAT, LAX, LBC, MCA, SAC, SBC, SDC, SFO, SPC; **South** (all ports on Gulf of Mexico, excluding FL): BRT, CCT, FTP, FTX, GAL, GMS, HOU, MAL, NOL, PAT, PIT; **Northeast** (all states north of and including NC): ABN, AMD, ANY, AVA, BAL, BAR, BAT, BNJ, BNY, BOO, BOS, BUF, CHI, CLY, CMA, CME, COH, DET, DMN, EME, ERI, GMA, GNY, HOL, JOL, KNY, MAC, MAR, MCI, MIL, MVY, NOR, NRI, NYC, OGS, OMA, OSW, PEJ, PHL, PHM, PME, PNH, PNY, PRI, PVM, RCK, RHI, RNY, SMA, SMI, STP, SYN, TCM, TNY, TRA, WDE, WNC, WPN, WRI, WVA, WYN, YRK; **Southeast** (all ports in FL, GA, and SC): CHA, JAX, KWE, MAN, MIA, NPF, PBF, PCF, PEN, PEV, SAV, SFL, SPF, TAM, VBF, WPB; **Caribbean Islands**: FPR, ISC, MAY, NYA, PPR, SJO, SJU, STC, STT.

**FIGURE 1:**
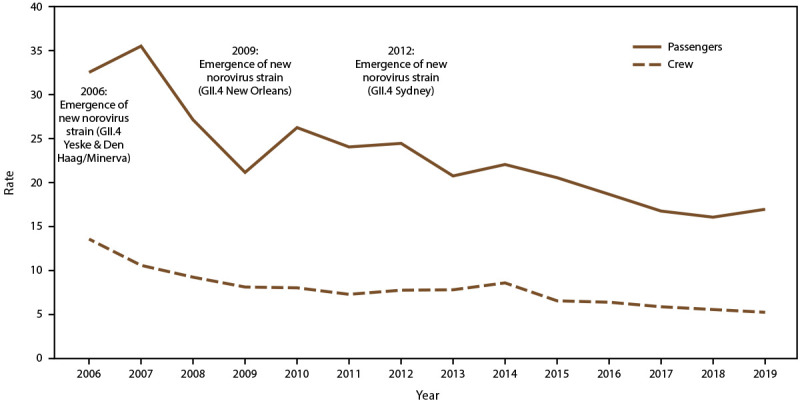
Incidence rate* of acute gastroenteritis on cruise ships, by year and traveler type — Maritime Illness Database and Reporting System, United States, 2006––2019^†^ * Per 100,000 travel days (defined as the sum of passengers/crew cases of the total number of voyage days). Rate = [(Total number of passenger/crew cases) / (total passengers/crew onboard x total number of voyage days during a voyage)] x 100,000 travel days. † Case counts are based on the last report submitted to Maritime Illness Database Reporting System and do not reflect final counts at the time of disembarkation; excludes ships with voyage length <3 and >21 days and <100 passengers or no crew.

During 2006–2019, incidence rates decreased significantly, from 32.5 cases per 100,000 travel days to 16.9 cases among passengers, and from 13.5 to 5.2 among crew (Figure 1). Among passengers, AGE incidence rates increased significantly with increasing ship size and voyages lasting more than 7 days, whereas for crew members, rates were significantly higher for mega (26.7 per 100,000 travel-days) and super-mega (29.2 per 100,000 travel days) ships compared with the smaller ship sizes and voyages >5 days compared with 3–5 day voyages (Table 2). Among crew, incidence rates were significantly higher for extra-large ships compared with other ship sizes and voyages lasting 6–7 days. Compared with rates on ships traveling to ports in the Southeast region, AGE incidence rates were higher on ships traveling to a port in California (32.0 per 100,00 travel days) or the South (23.7) among passengers and crew (24.8 and 25.9, respectively).

**TABLE 2:** Incidence rate* of acute gastroenteritis for passengers and crew, by cruise ship demographics and voyage characteristics — Maritime Illness Database and Reporting System, United States, 2006 – 2019

Characteristic	Total			Passengers		Crew	
	No.	Rate (95% CI)	p-value^†^	Rate (95% CI)	p-value	Rate (95% CI)	p-value
**Ship size (gross registered tons)^§^**							
Extra small, small, medium (≤30,000)	**1,500**	**9.06 (8.36–9.83)**	**<0.0001**	10.9 (9.94–12.1)	<0.0001	6.4 (5.52–7.45)	<0.0001
Large (30,001–60,000)	**4,510**	**21.4 (21.0–21.8)**	**0.0019**	23.7 (23.2–24.2)	0.0125	16.7 (16.1–17.4)	<0.0001
Extra large (60,001–120,000)	**30,039**	**22.1 (22.0–22.2)**	**Ref**	23.0 (22.9–23.1)	Ref	19.8 (19.6–20.0)	Ref
Mega (120,001–140,000)	**917**	**22.9 (22.4–23.4)**	**0.0007**	26.7 (26.1–27.4)	<0.0001	14.7 (14.0–15.4)	<0.0001
Super mega (≥140,001)	**292**	**24.4 (23.5–25.4)**	**<0.0001**	29.2 (27.8–30.5)	<0.0001	16.0 (14.8–17.4)	<0.0001
**Voyage length (days)^§^**							
3–5	**13,772**	**14.5 (14.3–14.6)**	**Ref**	13.3 (13.1–13.5)	Ref	17.5 (17.1–17.8)	Ref
6–7	**6,031**	**19.0 (18.7–19.2)**	**<0.0001**	17.8 (17.5–18.1)	<0.0001	22.1 (21.6–22.6)	<0.0001
8–10	**12,239**	**22.0 (21.8–22.2)**	**<0.0001**	23.2 (23.0–23.4)	<0.0001	19.0 (18.7–19.3)	<0.0001
11–14	**3,111**	**29.5 (29.2–29.9)**	**<0.0001**	35.0 (34.6–35.5)	<0.0001	17.4 (17.0–17.9)	0.9442
15–21	**2,105**	**33.8 (33.4–34.2)**	**<0.0001**	40.0 (39.5–40.5)	<0.0001	20.9 (20.4–21.5)	<0.0001
**Reporting port^¶^**							
California	**5,021**	**30.0 (29.6–30.3)**	**<0.0001**	32.0 (31.7–32.4)	<0.0001	24.8 (24.3–25.3)	<0.0001
Caribbean Islands	**2,267**	**20.5 (20.1–20.9)**	**<0.0001**	22.7 (22.2–23.2)	0.1906	15.5 (14.9–16.1)	<0.0001
Hawaiian Islands	**250**	**19.9 (18.7–21.1)**	**0.0151**	25.7 (24.4–27.0)	0.6176	12.8 (11.5–14.2)	<0.0001
Northeast	**3,756**	**20.4 (20.1–20.6)**	**<0.0001**	21.1 (20.7–21.4)	<0.0001	18.7 (18.2–19.2)	0.2261
Northwest	**3,384**	**14.7 (14.5–15.0)**	**<0.0001**	16.5 (16.1–16.8)	<0.0001	10.6 (10.2–11.0)	<0.0001
South	**2,767**	**24.3 (23.9–24.7)**	**<0.0001**	23.7 (23.2–24.1)	<0.0001	25.9 (25.1–26.7)	0.0201
Southeast	**19,813**	**21.4 (21.2–21.5)**	**Ref**	22.3 (22.1–22.5)	Ref	19.1 (18.8–19.3)	Ref

**Abbreviations:** CI = confidence interval; Ref = referent.

* [(Total no. passenger/crew cases) / (total passengers/crew onboard x total number of voyage days during a voyage)] x 100,000 travel days.

† Wald chi-square was used to assess the association between variables, with the significance threshold set at 0.05.

§ Categorization of ship size is based on Vessel Sanitation Program (VSP) categories used by VSP for the program’s construction and inspection activities, and voyage length is categorized to match cruise line marketing travel packages.

^¶^ **Northwest** (WA, OR, AK): ADK, AKU, ANC, AOR, ATT, BAK, BWA, COR, DHA, EFC, FHW, GLB, HAK, HNS, HOM, IAK, JNU, KIS, KOD, KTN, MET, NOM, PAN, POR, PTB, SEA, SGY, SIT, SWD, VDZ, WAI, WRG, WTR, YAK; **Hawaiian Islands, Guam, American Samoa, Saipan**: GUA, HIL, HNL, KAH, KAU, KON, LAH, MAU, PAS, SAI, SAM; **California**: ACA, CAT, LAX, LBC, MCA, SAC, SBC, SDC, SFO, SPC; **South** (all ports on Gulf of Mexico, excluding FL): BRT, CCT, FTP, FTX, GAL, GMS, HOU, MAL, NOL, PAT, PIT; **Northeast** (all states north of and including NC): ABN, AMD, ANY, AVA, BAL, BAR, BAT, BNJ, BNY, BOO, BOS, BUF, CHI, CLY, CMA, CME, COH, DET, DMN, EME, ERI, GMA, GNY, HOL, JOL, KNY, MAC, MAR, MCI, MIL, MVY, NOR, NRI, NYC, OGS, OMA, OSW, PEJ, PHL, PHM, PME, PNH, PNY, PRI, PVM, RCK, RHI, RNY, SMA, SMI, STP, SYN, TCM, TNY, TRA, WDE, WNC, WPN, WRI, WVA, WYN, YRK; **Southeast** (all ports in FL, GA, and SC): CHA, JAX, KWE, MAN, MIA, NPF, PBF, PCF, PEN, PEV, SAV, SFL, SPF, TAM, VBF, WPB; **Caribbean Islands**: FPR, ISC, MAY, NYA, PPR, SJO, SJU, STC, STT.

**FIGURE 2:**
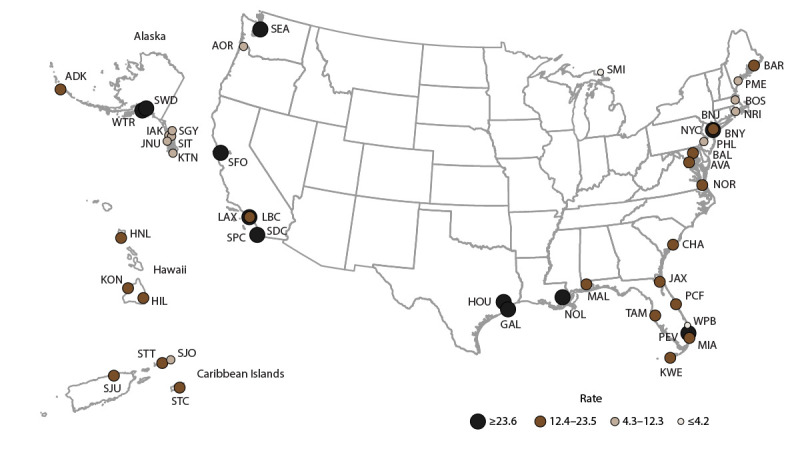
Incidence rate* of acute gastroenteritis reported by cruise ships anchoring at U.S. ports,**^†^** — Maritime Illness Database and Reporting System, United States, 2006–2019 * Per 100,000 travel days (defined as the sum of passengers/crew cases of the total number of voyage days). Rate = [(Total number of passenger/crew cases) / (total passengers/crew onboard x total number of voyage days during a voyage)] x 100,000 travel days. Rates (total population, passengers, and crew) were mapped using quartile breaks as cut points to measure the variability around the median rate. Ports with <20 reports were considered unstable and no data are presented. † **Northwest (WA, OR, AK):** ADK, AKU, ANC, AOR, ATT, BAK, BWA, COR, DHA, EFC, FHW, GLB, HAK, HNS, HOM, IAK, JNU, KIS, KOD, KTN, MET, NOM, PAN, POR, PTB, SEA, SGY, SIT, SWD, VDZ, WAI, WRG, WTR, YAK. **Hawaiian Islands, Guam, American Samoa, Saipan:** GUA, HIL, HNL, KAH, KAU, KON, LAH, MAU, PAS, SAI, SAM. **California:** ACA, CAT, LAX, LBC, MCA, SAC, SBC, SDC, SFO, SPC. **South (all ports on Gulf of Mexico, excluding FL):** BRT, CCT, FTP, FTX, GAL, GMS, HOU, MAL, NOL, PAT, PIT. **Northeast (all states north of and including NC):** ABN, AMD, ANY, AVA, BAL, BAR, BAT, BNJ, BNY, BOO, BOS, BUF, CHI, CLY, CMA, CME, COH, DET, DMN, EME, ERI, GMA, GNY, HOL, JOL, KNY, MAC, MAR, MCI, MIL, MVY, NOR, NRI, NYC, OGS, OMA, OSW, PEJ, PHL, PHM, PME, PNH, PNY, PRI, PVM, RCK, RHI, RNY, SMA, SMI, STP, SYN, TCM, TNY, TRA, WDE, WNC, WPN, WRI, WVA, WYN, YRK. **Southeast (all ports in FL, GA, and SC):** CHA, JAX, KWE, MAN, MIA, NPF, PBF, PCF, PEN, PEV, SAV, SFL, SPF, TAM, VBF, WPB. **Caribbean Islands:** FPR, ISC, MAY, NYA, PPR, SJO, SJU, STC, STT.

**FIGURE 3:**
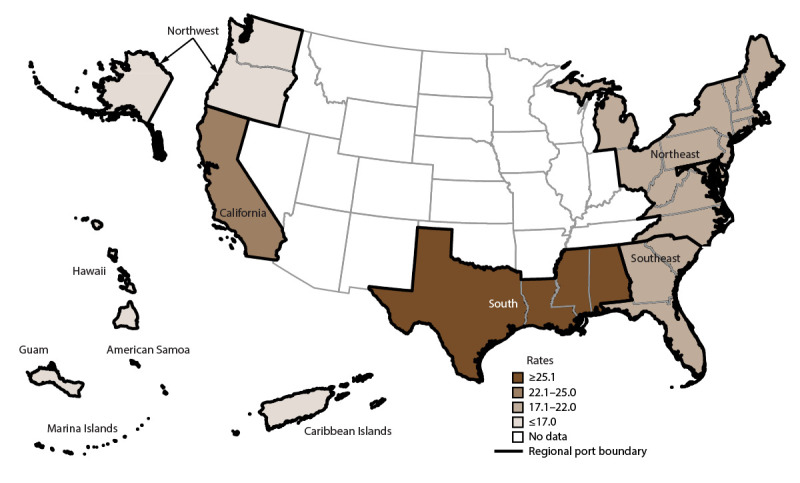
Incidence rate* of acute gastroenteritis among passengers, by U.S. regional port location,^†^ — Maritime Illness Database and Reporting System, United States, 2006–2019 * Per 100,000 travel days (defined as the sum of passengers/crew cases of the total number of voyage days). Rate = [(Total number of passenger/crew cases) / (total passengers/crew onboard x total number of voyage days during a voyage)] x 100,000 travel days. Rates (total population, passengers, and crew) were mapped using quartile breaks as cut points to measure the variability around the median rate. Ports with <20 reports were considered unstable and no data are presented. † **Northwest (WA, OR, AK):** ADK, AKU, ANC, AOR, ATT, BAK, BWA, COR, DHA, EFC, FHW, GLB, HAK, HNS, HOM, IAK, JNU, KIS, KOD, KTN, MET, NOM, PAN, POR, PTB, SEA, SGY, SIT, SWD, VDZ, WAI, WRG, WTR, YAK. **Hawaiian Islands, Guam, American Samoa, Saipan:** GUA, HIL, HNL, KAH, KAU, KON, LAH, MAU, PAS, SAI, SAM. **California:** ACA, CAT, LAX, LBC, MCA, SAC, SBC, SDC, SFO, SPC. **South (all ports on Gulf of Mexico, excluding FL):** BRT, CCT, FTP, FTX, GAL, GMS, HOU, MAL, NOL, PAT, PIT. **Northeast (all states north of and including NC):** ABN, AMD, ANY, AVA, BAL, BAR, BAT, BNJ, BNY, BOO, BOS, BUF, CHI, CLY, CMA, CME, COH, DET, DMN, EME, ERI, GMA, GNY, HOL, JOL, KNY, MAC, MAR, MCI, MIL, MVY, NOR, NRI, NYC, OGS, OMA, OSW, PEJ, PHL, PHM, PME, PNH, PNY, PRI, PVM, RCK, RHI, RNY, SMA, SMI, STP, SYN, TCM, TNY, TRA, WDE, WNC, WPN, WRI, WVA, WYN, YRK. **Southeast (all ports in FL, GA, and SC):** CHA, JAX, KWE, MAN, MIA, NPF, PBF, PCF, PEN, PEV, SAV, SFL, SPF, TAM, VBF, WPB. **Caribbean Islands:** FPR, ISC, MAY, NYA, PPR, SJO, SJU, STC, STT.

**FIGURE 4:**
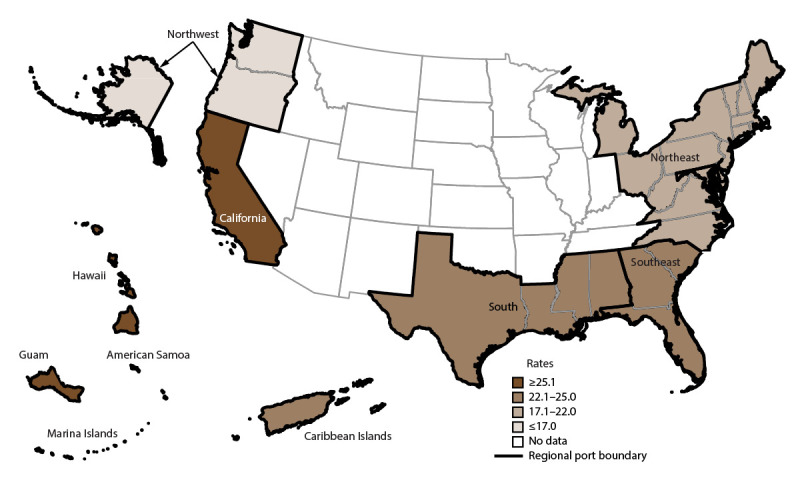
Incidence rate* of acute gastroenteritis among crew members, by U.S. regional port location,† — Maritime Illness Database and Reporting System, United States, 2006–2019 * Per 100,000 travel days (defined as the sum of passengers/crew cases of the total number of voyage days). Rate = [(Total number of passenger/crew cases) / (total passengers/crew onboard x total number of voyage days during a voyage)] x 100,000 travel days. Rates (total population, passengers, and crew) were mapped using quartile breaks as cut points to measure the variability around the median rate. Ports with <20 reports were considered unstable and no data are presented. † **Northwest (WA, OR, AK):** ADK, AKU, ANC, AOR, ATT, BAK, BWA, COR, DHA, EFC, FHW, GLB, HAK, HNS, HOM, IAK, JNU, KIS, KOD, KTN, MET, NOM, PAN, POR, PTB, SEA, SGY, SIT, SWD, VDZ, WAI, WRG, WTR, YAK. **Hawaiian Islands, Guam, American Samoa, Saipan:** GUA, HIL, HNL, KAH, KAU, KON, LAH, MAU, PAS, SAI, SAM. **California:** ACA, CAT, LAX, LBC, MCA, SAC, SBC, SDC, SFO, SPC. **South (all ports on Gulf of Mexico, excluding FL):** BRT, CCT, FTP, FTX, GAL, GMS, HOU, MAL, NOL, PAT, PIT. **Northeast (all states north of and including NC):** ABN, AMD, ANY, AVA, BAL, BAR, BAT, BNJ, BNY, BOO, BOS, BUF, CHI, CLY, CMA, CME, COH, DET, DMN, EME, ERI, GMA, GNY, HOL, JOL, KNY, MAC, MAR, MCI, MIL, MVY, NOR, NRI, NYC, OGS, OMA, OSW, PEJ, PHL, PHM, PME, PNH, PNY, PRI, PVM, RCK, RHI, RNY, SMA, SMI, STP, SYN, TCM, TNY, TRA, WDE, WNC, WPN, WRI, WVA, WYN, YRK. **Southeast (all ports in FL, GA, and SC):** CHA, JAX, KWE, MAN, MIA, NPF, PBF, PCF, PEN, PEV, SAV, SFL, SPF, TAM, VBF, WPB. **Caribbean Islands:** FPR, ISC, MAY, NYA, PPR, SJO, SJU, STC, STT.

**FIGURE 5:**
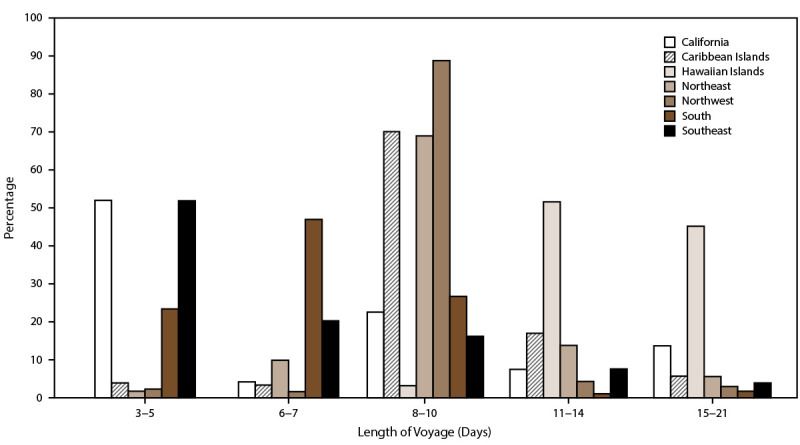
Percentage of acute gastroenteritis reports submitted, by regional port* and voyage length**^†^** — Maritime Illness Database and Reporting System, United States, 2006–2019 * **Northwest (WA, OR, AK):** ADK, AKU, ANC, AOR, ATT, BAK, BWA, COR, DHA, EFC, FHW, GLB, HAK, HNS, HOM, IAK, JNU, KIS, KOD, KTN, MET, NOM, PAN, POR, PTB, SEA, SGY, SIT, SWD, VDZ, WAI, WRG, WTR, YAK. **Hawaiian Islands, Guam, American Samoa, Saipan:** GUA, HIL, HNL, KAH, KAU, KON, LAH, MAU, PAS, SAI, SAM. **California:** ACA, CAT, LAX, LBC, MCA, SAC, SBC, SDC, SFO, SPC. **South (all ports on Gulf of Mexico, excluding FL):** BRT, CCT, FTP, FTX, GAL, GMS, HOU, MAL, NOL, PAT, PIT. **Northeast (all states north of and including NC):** ABN, AMD, ANY, AVA, BAL, BAR, BAT, BNJ, BNY, BOO, BOS, BUF, CHI, CLY, CMA, CME, COH, DET, DMN, EME, ERI, GMA, GNY, HOL, JOL, KNY, MAC, MAR, MCI, MIL, MVY, NOR, NRI, NYC, OGS, OMA, OSW, PEJ, PHL, PHM, PME, PNH, PNY, PRI, PVM, RCK, RHI, RNY, SMA, SMI, STP, SYN, TCM, TNY, TRA, WDE, WNC, WPN, WRI, WVA, WYN, YRK. **Southeast (all ports in FL, GA, and SC):** CHA, JAX, KWE, MAN, MIA, NPF, PBF, PCF, PEN, PEV, SAV, SFL, SPF, TAM, VBF, WPB. **Caribbean Islands:** FPR, ISC, MAY, NYA, PPR, SJO, SJU, STC, STT. † Voyage length is categorized to match cruise line marketing travel packages. Cruise ships sailing to Hawaiian islands are not represented in voyages of <8 days.

Overall, AGE incidence rates were highest among cruise ships visiting ports in California (San Diego, Los Angeles, San Pedro, and San Francisco), Alaska (Whittier and Seward), Texas (Houston and Galveston), New York (Brooklyn and New York City), Florida (Fort Lauderdale), and Louisiana (New Orleans) (Figure 2). Among passengers, AGE incidence rates were in the highest quartile for ships anchoring in California (32.1 per 100,000 travel days) (Figure 3); for crew, rates were in the highest quartile for ships anchoring in the South region (25.9) (Figure 4). The length of cruise ship voyages varied greatly depending on the regional port to which ships were traveling. The majority of ships traveling to ports in California and the Southeast region were on voyages lasting 3–5 days (52% and 51.9%, respectively); 6–7 days for ships anchoring in the South region (47.0%); 8–10 days for ships visiting ports in the Northwest (88.8%), Caribbean Islands (70.1%), and Northeast (68.7%) regions; and 11–21 days for ports in the Hawaiian Islands (97.0%) (Figure 5).

**FIGURE 6:**
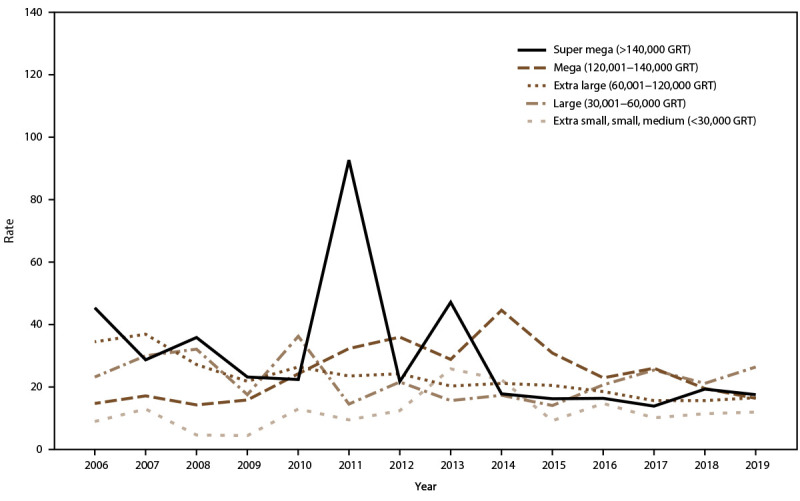
Incidence rate* of acute gastroenteritis among passengers, by ship size**^†^** and year — Maritime Illness Database and Reporting System, United States, 2006–2019 Abbreviation: GRT = gross registered tons. * Per 100,000 travel days (defined as the sum of passengers/crew cases of the total number of voyage days). Rate = [(Total number of passenger/crew cases) / (total passengers/crew onboard x total number of voyage days during a voyage)] x 100,000 travel days. † Categorization of ship size is based on the Vessel Sanitation Program categories used for the program’s construction and inspection activities.

**FIGURE 7:**
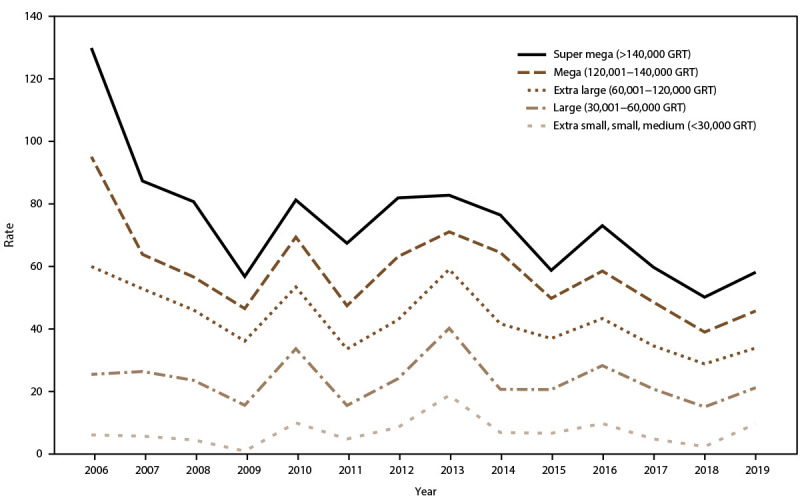
Incidence rate* of acute gastroenteritis among crew, by ship size**^†^** and year — Maritime Illness Database and Reporting System, United States, 2006–2019 Abbreviation: GRT = gross registered tons. * Per 100,000 travel days (defined as the sum of passengers/crew cases of the total number of voyage days). Rate = [(Total number of passenger/crew cases) / (total passengers/crew onboard x total number of voyage days during a voyage)] x 100,000 travel days. † Categorization of ship size is based on the Vessel Sanitation Program categories used for the program’s construction and inspection activities.

**FIGURE 8:**
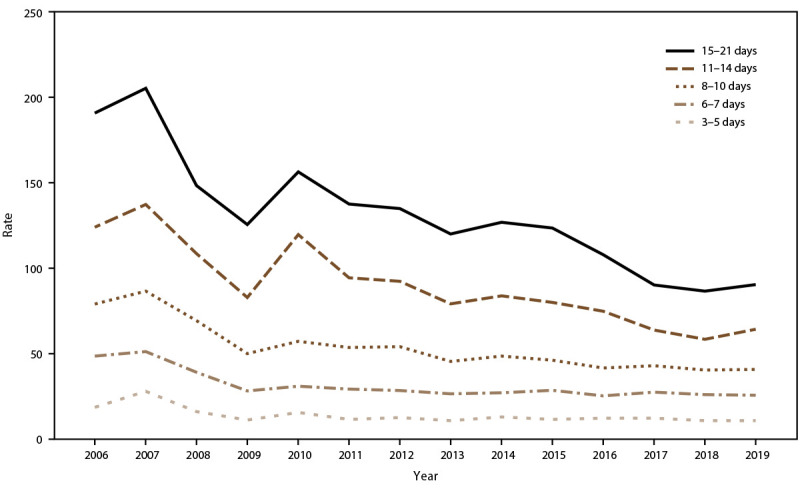
Incidence rate* of acute gastroenteritis among passengers, by voyage length**^†^** and year — Maritime Illness Database and Reporting System, United States, 2006–2019 * Per 100,000 travel days (defined as the sum of passengers/crew cases of the total number of voyage days). Rate = [(Total number of passenger/crew cases) / (total passengers/crew onboard x total number of voyage days during a voyage)] x 100,000 travel days. † Voyage length is categorized to match cruise line marketing travel packages.

**FIGURE 9:**
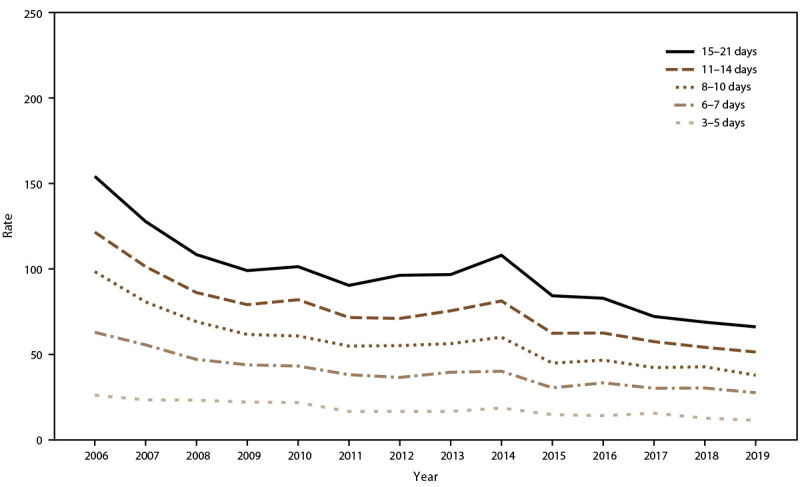
Incidence rate* of acute gastroenteritis among crew, by voyage length**^†^** and year — Maritime Illness Database and Reporting System, United States, 2006–2019 * Per 100,000 travel days (defined as the sum of passengers/crew cases of the total number of voyage days). Rate = [(Total number of passenger/crew cases) / (total passengers/crew onboard x total number of voyage days during a voyage)] x 100,000 travel days. † Voyage length is categorized to match cruise line marketing travel packages.

AGE incidence rate trends for passengers and crew were analyzed in relation to ship size and, during 2006–2019, rates showed a decreasing trend among extra-large, mega, and super-mega ships. Among passengers, AGE illness incidence rates peaked in 2010 and 2014 for mega ships, during 2011 and 2012 for super-mega ships, and in 2013 for small ships (≤30,000 GRT) (Figure 6). AGE incidence rates among crew increased in 2010, 2013, and 2016 for all ship sizes (Figure 7). Among passengers, rates remained relatively stable for voyages lasting <11 days. During 2006–2019, rates declined for voyages of 11–14 days and 15–21 days but remained higher than rates for voyages <11 days. Incidence rates spiked among passengers traveling on voyages lasting ≥6 days in 2007 and ≥11 days in 2010 (Figure 8). Among crew, annual incidence rates remained relatively stable regardless of voyage length, although in 2014 there was a slight increase in rates among cruise ships on voyages of 15–21 days (Figure 9).

### Outbreaks

**FIGURE 10:**
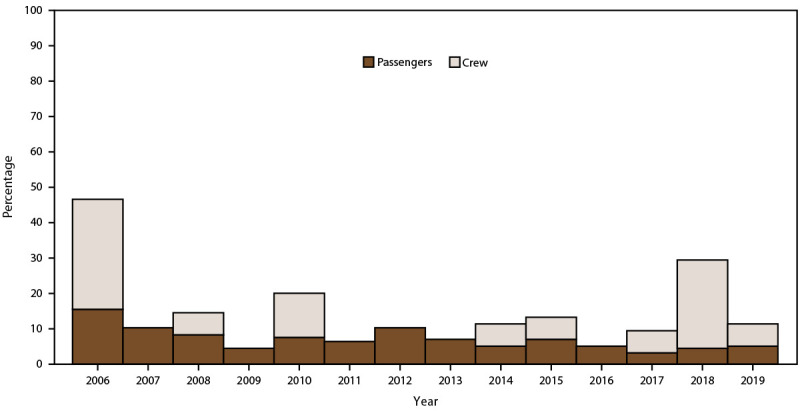
Percentage of acute gastroenteritis outbreaks among passengers and crew, by year — Maritime Illness Database and Reporting System, United States, 2006–2019* * During 2006–2019, VSP investigated 156 outbreaks among passengers and 16 outbreaks among crew.

During 2006–2019, VSP investigated 156 outbreaks among passengers and 16 outbreaks among crew. Of the 156 passenger-associated outbreaks, 63% occurred during 2006–2012; for crew, 50% occurred during 2014–2019 (Figure 10). A total of 117 (75%) outbreaks occurred among passengers on voyages lasting 11–21 days; for crew, 10 (63%) of 16 outbreaks occurred on voyages lasting 3–7 days (Table 3). A spatial cluster of 13 (81.3%) AGE outbreaks among crew was identified for cruise ships arriving at ports in the southeastern region. A total of 122 passenger outbreaks occurred on cruise ships visiting ports in the Southeast (44.3%), California (20.1%), and Northeast (17.5%) regions (Table 3).

**TABLE 3:** Number and percentage of acute gastroenteritis outbreak reports among passengers and crew, by voyage length and regional port location — Maritime Illness Database and Reporting System, United States, 2006–2019

Characteristic	Passengers (%)*	Crew (%)*
**Voyage length (days)^†^**		
3–5	7 (4)	6 (38)
6–7	2 (1)	4 (25)
8–10	30 (19)	3 (19)
11–14	57 (37)	2 (13)
15–21	60 (38)	1 (6)
**Regional port of arrival** ^§^		
California	30 (19)	0 (0)
Caribbean Islands	9 (6)	1 (6)
Hawaiian Islands	3 (2)	0 (0)
Northeast	27 (17)	1 (7)
Northwest	14 (9)	0 (0)
South	4 (3)	1 (6)
Southeast	66 (42)	13 (81)
Non-U.S.	3 (2)	0 (0)

* For passengers, N = 156; for crew, N = 16. Percentages might not total 100% because of rounding.

† Voyage length is categorized to match cruise line marketing travel packages.

§ **Northwest** (WA, OR, AK): ADK, AKU, ANC, AOR, ATT, BAK, BWA, COR, DHA, EFC, FHW, GLB, HAK, HNS, HOM, IAK, JNU, KIS, KOD, KTN, MET, NOM, PAN, POR, PTB, SEA, SGY, SIT, SWD, VDZ, WAI, WRG, WTR, YAK; **Hawaiian Islands, Guam, American Samoa, Saipan**: GUA, HIL, HNL, KAH, KAU, KON, LAH, MAU, PAS, SAI, SAM; **California**: ACA, CAT, LAX, LBC, MCA, SAC, SBC, SDC, SFO, SPC; **South** (all ports on Gulf of Mexico, excluding FL): BRT, CCT, FTP, FTX, GAL, GMS, HOU, MAL, NOL, PAT, PIT; **Northeast** (all states north of and including NC): ABN, AMD, ANY, AVA, BAL, BAR, BAT, BNJ, BNY, BOO, BOS, BUF, CHI, CLY, CMA, CME, COH, DET, DMN, EME, ERI, GMA, GNY, HOL, JOL, KNY, MAC, MAR, MCI, MIL, MVY, NOR, NRI, NYC, OGS, OMA, OSW, PEJ, PHL, PHM, PME, PNH, PNY, PRI, PVM, RCK, RHI, RNY, SMA, SMI, STP, SYN, TCM, TNY, TRA, WDE, WNC, WPN, WRI, WVA, WYN, YRK; **Southeast** (all ports in FL, GA, and SC): CHA, JAX, KWE, MAN, MIA, NPF, PBF, PCF, PEN, PEV, SAV, SFL, SPF, TAM, VBF, WPB; **Caribbean Islands**: FPR, ISC, MAY, NYA, PPR, SJO, SJU, STC, STT.

## Discussion

The incidence of reported AGE cases on cruise ships has declined over time, with higher rates observed among passengers compared with crew. The decline in AGE incidence rates might in part be associated with the evolution of environmental health sanitation standards that were established in 1975 by CDC and enforced during environmental health ship inspections conducted by VSP environmental health officers (*10*). Ship inspections were implemented in 1975 to ensure a higher level of shipboard sanitation to protect the health of the travelling public, including crew members who typically spend several months at a time onboard a cruise ship. In addition to implementation of ship inspections, cruise ship industry practices, such as hygiene standards, availability of hand hygiene stations in public areas throughout the ship, and health screening of passengers and crew before embarkation, also have likely contributed to the trend of declining AGE incidence rates. AGE incidence rates among passengers traveling on super-mega ships spiked in 2011. This finding might be attributed to changes in the AGE case definition. In 2011, the case definition was broadened to include the frequency of diarrheal episodes “above normal for the individual” to allow detection of “true” AGE cases as knowledge increased (*9*) (https://www.who.int/news-room/fact-sheets/detail/diarrhoeal-disease). Therefore, these changes should be considered when making inferences on AGE incidence rates during this time.

Variation in incidence rates and outbreaks between passengers and crew can be explained by differences in exposure sources and adherence to outbreak management plans. Norovirus is the leading cause of AGE and causes a substantial burden on cruise ships. In 2002, the number of norovirus illness outbreaks increased substantially both on cruise ships and on land (*11*) and, since 2006, approximately 90% of cruise ship outbreaks with known causative agents involved noroviruses (*12*). Norovirus is highly contagious and requires only a small inoculum to produce infection (<100 viral particles) (*13*). The predominant mode of transmission for norovirus is the fecal-oral route; however, it also can easily be transmitted by an infected person, including those without symptoms, through contaminated food, water, or environmental surfaces (*13*,*14*). Pathogens (i.e., norovirus) can be introduced into the cruise ship environment by ill passengers during embarkation (*14*,*15*); however, person-to-person transmission via ill cabin mates and public vomiting incidents contribute to the occurrence of most of the outbreaks among passengers (*16*). Although cruise ship outbreaks were described separately for passengers and crew, voyage outbreaks occur simultaneously among passengers and crew during a voyage and are not limited to a single population. Passenger- and crew-associated outbreaks were based on the outbreak threshold; thus, if ≥3% of passenger or crew populations experienced AGE symptoms at a given time, outbreaks were categorized as a passenger-associated outbreak or crew-associated outbreak.

Lower AGE incidence rates among crew compared with passengers might be a result of differences in exposure sources. Residential accommodations and life on cruise ships for crew are different from passengers because they maintain separate living, dining, and recreational facilities onboard the ship and have different embarkation and disembarkation areas. Many crew positions do not require direct contact with passengers. As a result, many crew members have limited interaction with passengers by using only crew designated areas and interact with the same crew members for the length of their contracts. In addition, cruise companies have more control over crew to enforce adherence to public health measures and policies for preventing and mitigating AGE transmission, such as proper/frequent handwashing, prompt reporting of AGE symptoms, and adherence to isolation policies. For example, crew are required to promptly report illness at the time of onset and follow isolation procedures or face disciplinary actions for violating illness prevention and control guidelines. For passengers, cruise ship personnel have less authority to enforce adherence to illness prevention and control guidelines. As a result, nonreporting or delayed reporting of illness and noncompliance with isolation instructions among passengers is an ongoing problem that increases the risks for illness transmission and outbreaks.

The findings in this report indicate that incidence rates varied by ship size, voyage length, and regional port location. Overall, higher rates were associated with increasing ship size. Each year, new cruise ships are built to accommodate the growing number of cruisers (*17*). Since 1995, VSP has published public health guidelines for the construction and design of new and renovated cruise ships to promote uniform construction standards to meet sanitary criteria and limit the introduction and transmission of communicable diseases (https://www.cdc.gov/nceh/vsp/docs/vsp_construction_guidelines_2018-508.pdf). In addition, VSP partners with shipyards and vessel owners during plan reviews, consultations, and construction inspections to ensure public health safety and limit the introduction and transmission of communicable diseases onboard densely populated passenger cruise ships. The findings in this report also indicate that AGE incidence rates are higher on cruises lasting >7 days and that rates vary by regional port location. This report documents higher rates on cruise ships traveling to ports in California and lower rates in the Northwest region, whereas results in a previous study indicate rates to be highest in the Northwest region and lowest in the Northeast (*18*). Differences in findings might be due to the time frame in which the studies were conducted and correlation with the emergence of new norovirus strains (*14*,*19*). When stratified by voyage length, a greater percentage of cruise ships anchoring in California had voyages lasting 3–5 days. Voyages lasting 3–7 days result in high turnover of passengers, leading to a greater opportunity to expose crew to ill passengers, especially in regions where prevalence of illness is high. In addition to higher rates, these findings also indicate that AGE outbreaks are more frequent on voyages lasting >7 days among passengers and 6–7 days among crew. Voyages lasting >7 days present greater opportunity for person-to-person transmission because of extended time in a semienclosed environment. Prompt reporting of AGE symptoms is optimal for preventing outbreaks. In 2017, U.S. passengers accounted for 46% of global cruise ship travelers (26.8 million passengers), and the number of transit passengers visiting U.S. ports increased by 8% from 2016 to 2017 (5.6 million passengers) (*20*). With millions of passengers visiting U.S. ports, delayed reporting of AGE symptoms by passengers also could affect the health of shoreside communities and residents.

## Limitations

The findings in this report are subject to at least four limitations. First, incidence rates should be interpreted as an underestimation of actual maritime AGE incidence rates because final AGE case counts for voyages that did not meet or exceed the outbreak threshold are not reported to VSP, voyages lasting >15 days are not required to report cases that occurred prior to 15 days of arrival to the United States, and cases are not reported if persons did not seek medical care onboard the ship. Second, the estimated incidence for voyages lasting 3–7 days might be biased because exposure to norovirus on these shorter voyages might result in symptoms occurring after the voyage has ended, leading to underreporting of AGE cases. Third, findings might be affected by reporting bias because not all symptomatic passengers report their symptoms to the medical center, and diagnoses of symptomatic cases are not validated with medical records or laboratory findings. In addition, standardized procedures are needed to retract reports sent incorrectly or with erroneous case counts; however, ongoing procedures are used to ensure information is as accurate as possible. For example, limited error checking is embedded in the web-based portal to MIDRS to validate case counts for passengers and crew to minimize reporting errors. Finally, the case definition for AGE illness was broadened in 2011 with the goal of identifying additional cases of AGE so cruise ship medical crew could treat, isolate, and monitor persons who are at risk for exposing others, thus limiting the spread of illness onboard ships. The case definition change in 2011 caused a spike in incidence rates and might have captured other illnesses.

## Future Directions

This report examines the incidence of maritime AGE by cruise ship demographics and voyage characteristics. Accurate health monitoring of shipboard populations and improving incidence estimates rely on precise reporting of AGE cases. MIDRS data do not represent final AGE case counts at the time of disembarkation. Final voyage AGE case counts are not required to be reported to MIDRS, likely leading to an underestimation of the actual incidence of AGE illness and limiting accurate trend analysis, which can hamper efforts to improve public health interventions and policies to reduce disease transmission. Strengthening surveillance of AGE incidence would require both changes in AGE case count reporting policies and cruise ships to submit an end-of-voyage AGE case report.

Examination of voyage itineraries and land-based activities might be helpful to better understand disproportionate rates of AGE illness across U.S. regional ports. Additional research and evaluation are needed to determine possible causal relations between delayed reporting of AGE illness to ship medical personnel and demographic characteristics of traveling passengers. Another area for continued work includes cruise ship companies establishing new methods for prompt identification of ill passengers who do not report AGE symptoms or comply with isolation instructions during the infectious period.

## Conclusion

Millions of passengers travel on cruise ships from U.S. ports every year. Since 1975, VSP has monitored AGE illness and conducted environmental health sanitation inspections of hundreds of cruise ship voyages each year. Ongoing public health prevention efforts to identify and eliminate risks for AGE illness associated with environmental contamination, person-to-person, and food and waterborne pathogen transmission are essential for reducing AGE incidence rates among passengers and crew. Continued surveillance provides an opportunity for VSP to work collaboratively with cruise lines to reduce the risk for AGE transmission onboard and mitigate possible transmission to local communities.

Promotion of proper handwashing practices is an effective strategy for preventing AGE outbreaks (*21*). Cruise ship travelers should adhere to recommendations for handwashing and sanitation during cruise ship travel and promptly report gastrointestinal illness symptoms to medical personnel (including mild symptoms) and follow isolation instructions. Federal and state programs to reduce the incidence of AGE illness in communities across the United States should consider innovative messaging for informing travelers about the risk for AGE and the importance of their role in minimizing their risk for illness while traveling onboard passenger cruise ships, especially for persons who are most vulnerable to infection. Health care providers should consider discussing the importance of good personal hygiene and proper handwashing during clinical examinations with patients, especially those planning to take a cruise.
